# Ultrasensitive Electrochemical Detection and Plasmon-Enhanced Photocatalytic Degradation of Rhodamine B Based on Dual-Functional, 3D, Hierarchical Ag/ZnO Nanoflowers

**DOI:** 10.3390/s22135049

**Published:** 2022-07-05

**Authors:** Neethu Sebastian, Wan-Chin Yu, Deepak Balram

**Affiliations:** 1 Institute of Organic and Polymeric Materials, National Taipei University of Technology, No. 1, Section 3, Zhongxiao East Road, Taipei 106, Taiwan; neethusebastian6@gmail.com; 2 Department of Electrical Engineering, National Taipei University of Technology, No. 1, Section 3, Zhongxiao East Road, Taipei 106, Taiwan; deepubec2007@gmail.com

**Keywords:** electrochemical sensor, voltammetry, synthetic dye, photocatalysis, zinc oxide

## Abstract

The sensitive detection and degradation of synthetic dyes are pivotal to maintain safety owing to the adverse side effects they impart on living beings. In this work, we developed a sensitive electrochemical sensor for the nanomolar-level detection of rhodamine B (RhB) using a dual-functional, silver-decorated zinc oxide (Ag/ZnO) composite-modified, screen-printed carbon electrode. The plasmon-enhanced photocatalytic degradation of organic pollutant RhB was also performed using this nanocomposite prepared by embedding different weight percentages (1, 3, and 5 wt%) of Ag nanoparticles on the surface of a three-dimensional (3D), hierarchical ZnO nanostructure based on the photoreduction approach. The structure and morphology of an Ag/ZnO nanocomposite were characterized by scanning electron microscopy (SEM), transmission electron microscopy (TEM), elemental mapping, ultraviolet-visible (UV-vis) spectroscopy, and X-ray diffraction (XRD). The electrochemical sensor exhibited a very high sensitivity of 151.44 µAµM^−1^cm^−2^ and low detection limit of 0.8 nM towards RhB detection. The selectivity, stability, repeatability, reproducibility, and practical feasibility were also analyzed to prove their reliability. Furthermore, the photocatalysis results revealed that 3 wt% of the Ag/ZnO hybrid nanostructure acquired immense photostability, reusability, and 90.5% degradation efficiency under visible light. Additionally, the pseudo-first-order rate constant of Ag-3/ZnO is 2.186 min^−1^ suggested promising activity in visible light photocatalysis.

## 1. Introduction

Rhodamine B (9-(2-Carboxyphenyl)-6-(diethylamino)-N,N-diethyl-3H-xanthen-3-iminium chloride (RhB)) is a hydrophilic, bright-pink synthetic dye widely used in industries such as pharmaceuticals, cosmetics, printing, textiles, etc. As RhB possesses a relatively high stability and is inexpensive, it is widely used as a coloring agent in food industries to make foods appetizing to consumers. However, the toxic nature of RhB and adverse side effects are concerning factors [1,2]. As a consequence, its usage in food products is restricted in different countries [3]. Despite various regulations, RhB is still used as a coloring agent in food industries. Hence, the accurate detection of this mutagenic dye in food is important. Different analytical methods such as spectrophotometry [4], immunosorbent assay [5], surface enhanced Raman spectroscopy [6], and chromatography [7] were used for detecting RhB. In this work, we used a low-cost, faster, less complex, and sensitive electrochemical method for the nanomolar detection of RhB. Moreover, many of the synthetic dyes, notably the ones with complex aromatic and heterocyclic structures, are non-biodegradable and can ultimately find their way into the environment, especially when the wastewater is not adequately treated [8,9,10]. This shows the importance of the efficient photocatalytic degradation of RhB. Hence, along with electrochemical detection, RhB degradation was also performed using a dual-functional nanomaterial in this work. 

Heterogeneous photocatalysis is an advanced oxidation process (AOP) that generates reactive oxygen species (ROS) with the aid of semiconductor photocatalysts [11,12,13,14,15]. Particularly, zinc oxide (ZnO) has received great attention due to its favorable characteristics including large availability, non-toxicity, good mechanical strength, excellent biocompatibility, high thermal stability, antifouling, and antibacterial abilities [16,17]. The efficiency of a photocatalyst mainly depends on its ability to produce photoinduced charge carriers and the band edge energy level of the charge carriers, which are determined by the morphology, size, surface area, and optical properties of the photocatalyst. ZnO is available in various morphologies including nanowires, needles, nanorods, nanosheets, and nanoflowers [18,19,20]. Considering all the different morphologies, three-dimensional (3D) nanosheet-based ZnO exhibits superior performance for the degradation of organic pollutants. ZnO nanoparticles (NPs) have been used in the development of electrochemical sensors, also due to their excellent properties [21,22,23,24]. For instance, Earth-abundant ZnO maintains stability at high isoelectric points, and its outstanding binding property aids efficient molecule detection. Based on the aforementioned facts, it is known that ZnO nanomaterials can be considered for application in both photocatalysis and electrochemical applications. However, a swift recombination of charge carriers and the inability to absorb visible light are the main drawbacks of ZnO. Researchers have performed doping, defects’ engineering, morphology tuning, hybridization, and so on to modify the photocatalysts [25]. In this scenario, an effective way to address the drawbacks of using ZnO as a photocatalyst was to combine ZnO with noble metal NPs to form metal-metal oxide interfaces [26,27].

In this work, the electrochemical detection and photocatalytic degradation of RhB using Ag-3/ZnO is reported. Photoreduction was used to embed different weight percentages of Ag NPs on 3D hierarchical ZnO prepared using an aqueous solution approach. Various electrochemical investigations based on cyclic voltammetry (CV) and differential pulse voltammetry (DPV) techniques were performed to evaluate the electrocatalytic activity of an Ag-3/ZnO-modified, screen-printed carbon electrode (SPCE) towards RhB determination. As per our best knowledge, this is the first work that reports electrochemical detection and degradation of RhB using a dual-functional nanomaterial. Moreover, no works have yet reported on the electrochemical detection of RhB using Earth-abundant and low-cost ZnO. In the forthcoming sections, a nanomaterial preparation and an analysis of their characterizations are detailed. Discussions about the results from electrochemical experiments and photocatalytic degradation of RhB are also provided in this paper.

## 2. Materials and Methods

### 2.1. Materials

Zinc nitrate hexahydrate [Zn(NO_3_)_2_·6H_2_O] and sodium citrate dihydrate (HOC(COONa)(CH_2_COONa)_2_·2H_2_O) were obtained from J.T Baker (New Taipei City, Taiwan). Sodium hydroxide (NaOH), silver nitrate (AgNO_3_), and RhB were received from Sigma Aldrich (New Taipei City, Taiwan). All the chemicals were used without further purification. The chemicals used in this study were of analytical grade, and all the required solutions were prepared using deionized water. 

### 2.2. Instrumentation

The X-ray crystallographic study was conducted with a Bruker D8 Advance diffractometer and the morphological analysis was by a Hitachi S-4800 microscopy operated at 15 kV. A JEOL 1010 at 100 kV was used to obtain the transmission electron micrograms. The elemental mapping of the Ag/ZnO nanohybrid was evaluated by a JEOL JSM-7610F FE-SEM. A Jasco V-770 spectrophotometer was utilized for analyzing the optical properties and the photodegradation study of the as-prepared samples. All the electrochemical studies were performed using Metrohm Autolab. A three-electrode system with Ag/AgCl as a reference electrode, a platinum wire as a counter electrode, and the prepared nanocomposite-modified SPCE as a working electrode was used for the electrochemical experiments. 

### 2.3. Synthesis of 3D, Hierarchical ZnO Nanoflowers

The direct precipitation method was used for synthesizing hierarchical ZnO nanoflowers. As in the typical synthesis method, 1 M Zn(NO_3_)_2_·6 H_2_O, 4 M NaOH, 1 M C_6_H_5_Na_3_O_7_, and 2 H_2_O were dissolved in 20 mL, 30 mL, and 10 mL of distilled water independently. The above three solutions were kept in an ice bath for 20 min, and the temperature was strictly maintained below 5 °C. The above three solutions were mixed together. A white precipitate occurred first; it disappeared after stirring of 1 min. The mixed solution was added slowly to the 500 mL of aqueous solution under magnetic stirring. The reaction was continued for 1 h; the resultant white-color mixture was centrifuged, washed, and dried at 60 °C overnight.

### 2.4. Synthesis of Ag-Modified ZnO Nanoflowers Based on Photoreduction Method

The photoreduction method was used for the Ag/ZnO nanocomposite preparation. In the process, 1 g of ZnO was dispersed in 10 mL water, and a certain quantity (0.01 g, 0.03 g, and 0.05 g) of AgNO_3_ was weighed. Then AgNO_3_ was transferred into the ZnO dispersion under stirring. The mixture was magnetically stirred in the dark for 1 h. After continuous stirring in the dark, the mixture was taken for the irradiation of UV light for 30 min under stirring. Finally, it was centrifuged, washed, and dried at 60 °C for 12 h. We synthesized various concentrations, of 1, 3, and 5 wt%, of Ag-modified ZnO and named them as Ag-1/ZnO, Ag-3/ZnO, and Ag-5/ZnO, respectively.

### 2.5. Preparation of Ag-3/ZnO-Modified, Screen-Printed Carbon Electrode

For the fabrication of Ag-3/ZnO-modified SPCE (Ag-3/ZnO/SPCE), primarily 0.005 g Ag-3/ZnO nanomaterial was dispersed in 5 mL of water. The resultant solution was sonicated for 1 h to attain a homogeneous dispersion. Then, 8 µL of the dispersed nanomaterial solution was carefully drop casted on the surface of the SPCE. Then, it was dried at room temperature. The prepared Ag-3/ZnO/SPCE was then utilized for the electrochemical experiments carried out in this work.

### 2.6. Photocatalytic Degradation Study

The photocatalytic performance of ZnO and Ag-modified ZnO was analyzed by conducting RhB degradation in a photocatalytic reactor under visible light. In the experiment, a 50 mg catalyst was immersed in 100 mL of aqueous solution containing 1 × 10^−5^ M RhB. Then, sonication was conducted for attaining an adsorption–desorption equilibrium followed by visible light irradiation. The degradation characteristics were each examined for 30 min by fetching aliquots (3 mL) of RhB taken from the photocatalytic reactor. These aliquots were centrifuged to remove inorganic particles; the resultant supernatant solution was taken for the absorption studies in a UV/vis spectrometer, ranging from 200–800 nm. The degradation efficiency (*η*) of the dye solution is illustrated below [28].

(1) $$\eta =\;\frac{C_{0}-\;C_{t}}{C_{0}}\;\times 100$$

where *C*_0_ is the initial dye concentration and *C_t_* is the dye concentration after time *t* (minutes).

## 3. Results and Discussion

### 3.1. Characterizations

**Figure 1:**
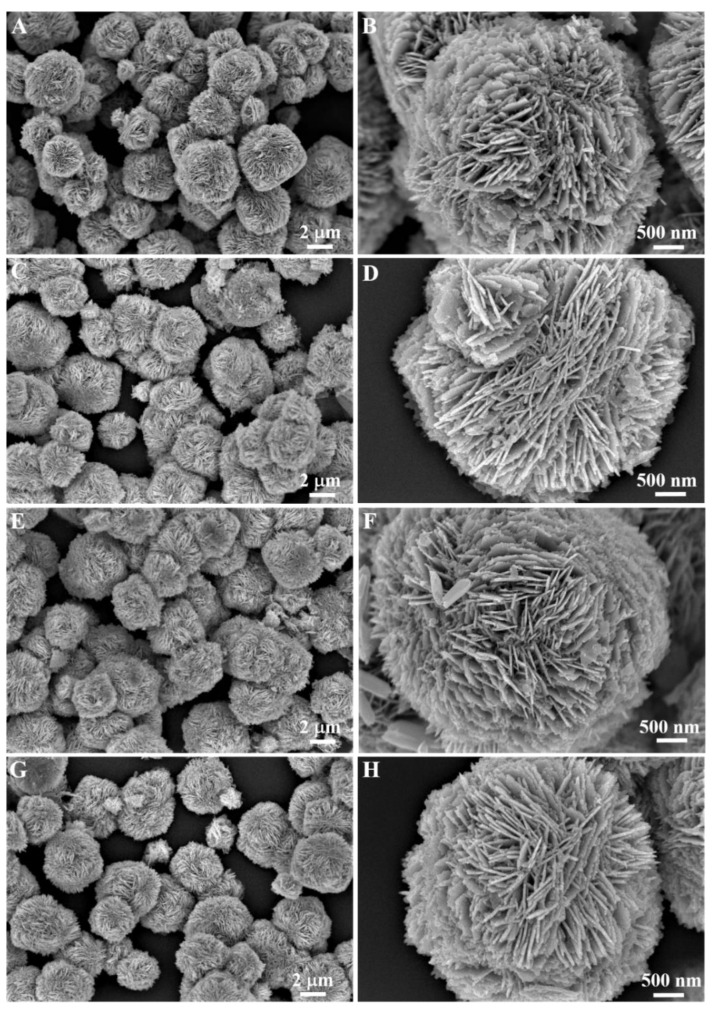
SEM images of ZnO nanoflowers (**A**,**B**), Ag-1/ZnO (**C**,**D**), Ag-3/ZnO (**E**,**F**), and Ag-5/ZnO (**G**,**H**).

The morphology of the prepared nanocomposite was performed using scanning electron microscopy (SEM) and transmission electron microscopy (TEM) in this work. Figure 1 reveals the SEM images of pure ZnO and Ag-modified (1%, 3%, and 5%) ZnO. According to the SEM images, pure ZnO depicted 3D, hierarchical nanosheet-based, flower-like nanostructures with diameters of 2–4 µm. When we analyzed the high-resolution view of the pure ZnO nanostructure, shown in Figure 1B, we observed that the 3D, flower-like nanostructures were formed by a number of self-assembled two-dimensional (2D) nanosheets with the thickness of 10–20 nm. The morphologies of Ag-1/ZnO, Ag-3/ZnO, and Ag-5/ZnO are depicted in Figure 1C–H; it can be noticed that the ZnO morphology remained the same even after the modification of Ag NPS. Ag-modified ZnO also showcased the 3D, nanosheets-based, spherical, hierarchical structure with diameters of 2–4 µm. 

**Figure 2:**
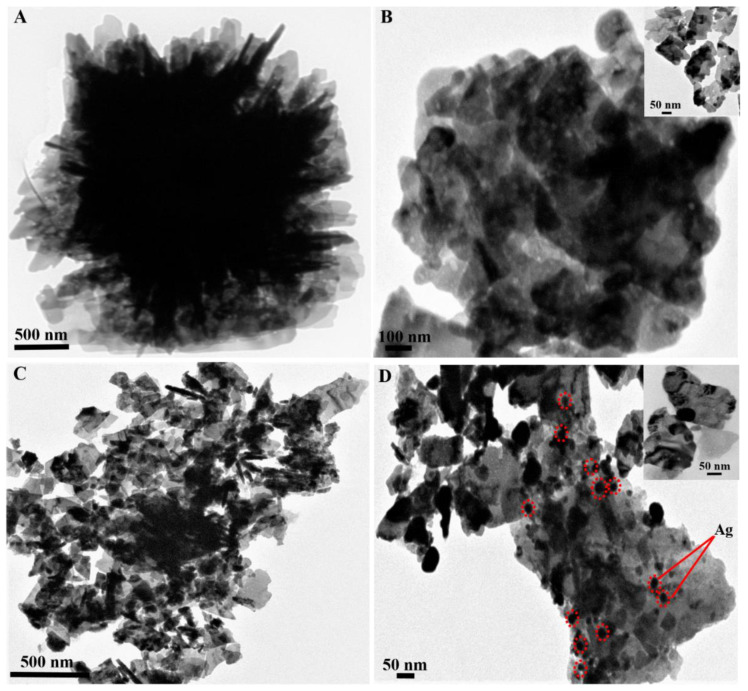
TEM images of 3D, hierarchical, nanosheet-based, flower-like ZnO (**A**,**B**) and Ag-3/ZnO (**C**,**D**). The red dot circle indicates Ag nanoparticles.

**Figure 3:**
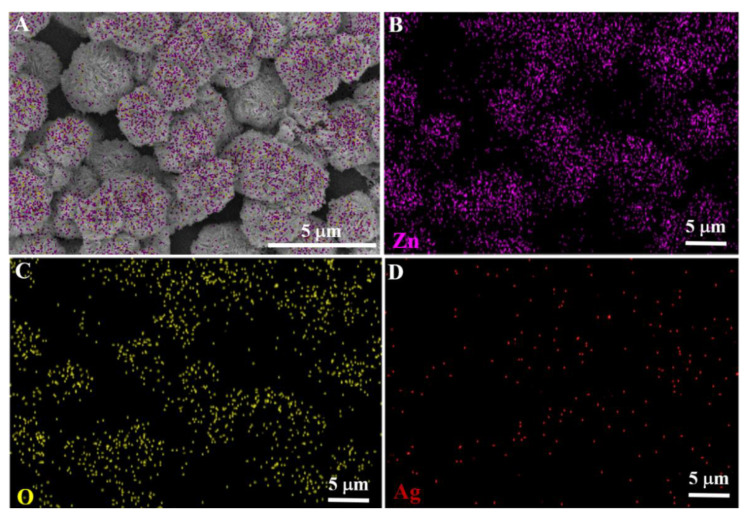
SEM of Ag-3/ZnO (**A**); corresponding elemental mapping of zinc (**B**), oxygen (**C**), and silver (**D**).

The TEM results of pure the ZnO and Ag/ZnO nanostructures are depicted in Figure 2. It is apparent that pure ZnO possessed a spherical, nanosheet-based morphology with diameters ranging from 2–4 µm, consistent with the SEM results. The spherical ZnO nanostructures consisted of numerous agglomerated nanosheets with an average length of 200–400 nm, which are clearly visible from the inset of Figure 2B. The TEM images of the Ag/ZnO also exhibited a spherical, nanosheet-based structure with the same size as the pure ZnO. As shown in Figure 2C,D, Ag NPs were located on the nanosheets’ surface, approximately 30–60 nm in size. The TEM results suggested the successful decoration of Ag NPs on the 3D, hierarchical ZnO nanostructures. Furthermore, the elemental mapping images of the Ag/ZnO depicted in Figure 3 confirmed the existence of Zn, O, and Ag elements.

**Figure 4:**
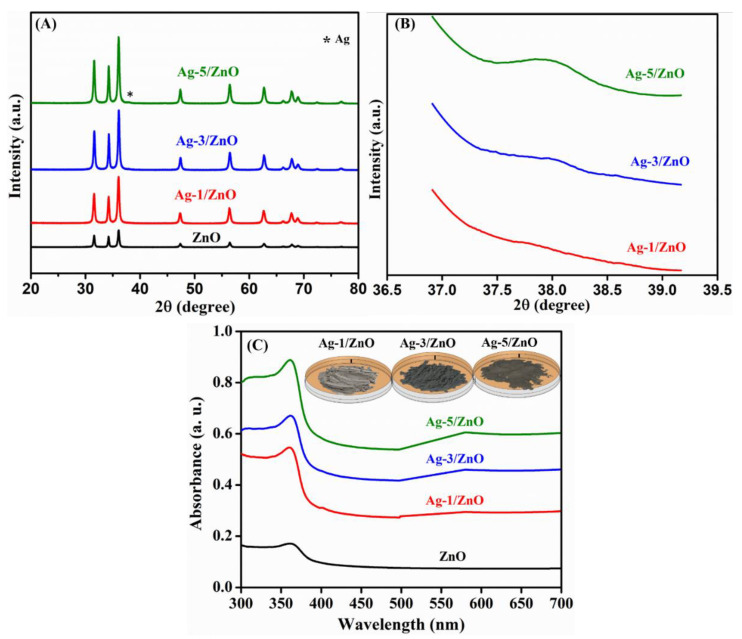
The XRD pattern: as-prepared samples (**A**) and UV-vis absorption spectra (**B**,**C**) of pure ZnO, Ag-1/ZnO, Ag-3/ZnO, and Ag-5/ZnO.

The crystalline structure and phase purity of Ag-modified ZnO and pure ZnO were determined by X-ray diffraction (XRD). Figure 4 shows the XRD patterns for ZnO, Ag-1/ZnO, Ag-3/ZnO, and Ag-5/ZnO. All prepared samples displayed intense and sharp diffraction peaks; pure ZnO exhibited all diffraction peaks identical to a wurtzite hexagonal structure of ZnO (JCPDS no. 36–1451) [29]. In addition, the diffraction peak of the pure ZnO nanomaterial appeared at 2θ = 31.7°, 34.5°, 36.3°, 47.4°, 56.6°, 62.7°, 66.4°, 67.9°, 69.1°, 72.5°, and 77.2°, corresponding to the (1 0 0), (0 0 2), (1 0 1), (1 0 2), (1 1 0), (1 0 3), (2 0 0), (1 1 2), (2 0 1), (0 0 4), and (2 0 2) planes, respectively. No other diffraction peaks were obtained in the XRD data, indicating the purity of the prepared samples. The XRD of Ag-1/ZnO, Ag-3/ZnO, and Ag-5/ZnO also exhibited similar types of diffraction peaks as the pure ZnO, which proved the formation of a hexagonal wurtzite structure of ZnO. Ag-5/ZnO attained a new diffraction peak at 37.92°, which was attributed to the (111) plane of the face-centered, cubical Ag. The appearance of Ag peaks (Figure 4B) implied the origination of crystalline Ag NPs on the 3D, hierarchical ZnO surface. The absence of this peak in Ag-1/ZnO and Ag-3/ZnO might be due to the low concentration of the Ag content deposited on the nanocomposite.

The absorbance spectrum of ZnO, Ag-1/ZnO, Ag-3/ZnO, and Ag-5/ZnO are depicted in Figure 4C. All four samples exhibited a well-defined absorption band at 365 nm. The absorption of the pure ZnO was obtained only in the UV region, indicating the inability of light absorption in the visible region, whereas the Ag-1/ZnO, Ag-3/ZnO, and Ag-5/ZnO composites exhibited a slight red shift and obtained an expansion of more intense absorption from the UV to visible region due to the surface plasmon resonance effect created by the Ag. Moreover, the intensity of the visible region absorbance was enhanced with the increase in Ag on the ZnO nanostructure; the highest absorption intensity was depicted by the Ag-5/ZnO nanocomposite.

### 3.2. Electrochemical Investigations for RhB Detection

**Figure 5:**
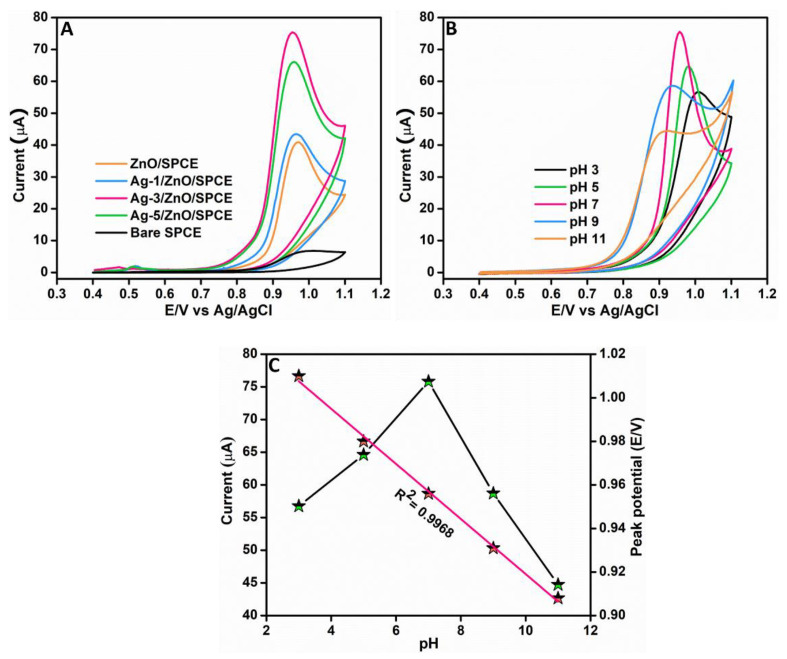
Voltammetric curves of different modified electrodes in 0.05 M PBS (pH 7.0) in presence of 100 μM RhB at 0.1 V/s (**A**). Voltammetric curves of Ag-3/ZnO/SPCE at different pH from pH 3 to pH 11 in presence of 100 μM RhB at 0.1 V/s (**B**). The pH against peak current and potential (**C**).

An experiment was conducted using different modified electrodes for evaluating the electrocatalytic activity of the prepared nanocomposite towards RhB detection during the initial phase of this electrochemical study. CV was utilized for analyzing the performance of modified electrodes including pure ZnO/SPCE, Ag-1/ZnO/SPCE, Ag-3/ZnO/SPCE, Ag-5/ZnO/SPCE, and bare SPCE. Figure 5A depicts the resultant voltammetric responses attained for all the modified electrodes we considered in this experiment. This comparative evaluation experiment was carried out in 0.05 M phosphate buffer solution (PBS) (pH 7) in the existence of 100 μM RhB at a scan rate of 0.1 V/s. The comparatively weakest voltammetric response was achieved by the bare SPCE; the resultant low value for peak current corresponded to its inferior electrocatalytic activity. Further, when we evaluate the voltammetric response of the pure ZnO/SPCE in Figure 5A, we can notice an anodic peak with a peak potential of 0.97 V. The voltammetric responses of the Ag-1/ZnO/SPCE and Ag-5/ZnO/SPCE also resulted in anodic peaks with a peak potential of 0.96 V and better current response than the pure ZnO/SPCE. However, the voltammetric response of the Ag-3/ZnO/SPCE towards RhB detection resulted in an anodic peak with a comparatively lowest peak potential of 0.95 V. Moreover, it was the Ag-3/ZnO/SPCE that showed a comparatively better voltammetric response towards RhB detection with a highly intense oxidation peak current, which was 11 times that of the bare SPCE. This showed the outstanding electrochemical irreversible oxidation of RhB, which is a 2-electron, 1-proton transfer process. The decoration of the Ag NPs on the ZnO aided in bringing the detection targets (RhB) onto the ZnO surface by a specific molecular interaction and widened its applicability in the detection of RhB. Therefore, it was evident that the Ag-3/ZnO/SPCE is apt for the electrochemical detection of RhB. 

#### 3.2.1. Optimization of pH towards RhB Determination

The pH of a solution has a significant role in the electrochemical process; hence, it is a critical factor to be scrutinized in electrochemical investigations. As part of the study, an experiment was conducted to analyze the pH impact in RhB determination using the Ag-3/ZnO/SPCE and to ultimately determine the optimal pH for the electrochemical determination of RhB. This experiment was carried out in 0.05 M PBS containing 100 μM RhB under different pH at a scan rate of 0.1 V/s. Figure 5B shows the voltammetric response we obtained by carrying out this experiment in various pH of an electrolyte. The analysis of the voltammetric curves showed that the oxidation peak potential decreased with an increase in the pH; the lowest oxidation peak potential 0.9 V was achieved at pH 11 and the highest, 1.01 V, at pH 3. Moreover, peak currents increased from pH 3 to pH 7 and decreased from pH 7 to pH 11. Hence, it was understood that the strongest oxidation peak currents can be obtained when the analyte pH is maintained at 7. The calibration curve of pH against anodic peak current and peak potential is shown in Figure 5C; the resultant regression equation is *E*_pa_ (V) = −0.01 pH + 1.04 (R^2^ = 0.9968). From the aforementioned inferences from this experiment, pH 7 was selected as the optimal pH for the electrochemical detection of RhB.

#### 3.2.2. Effect of Concentration and Scan Rate

**Figure 6:**
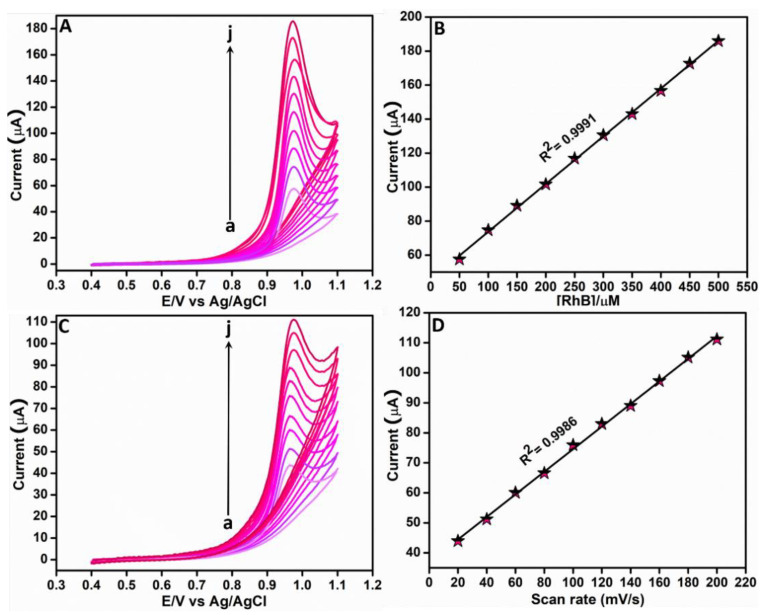
(**A**) Voltammetric curves of Ag-3/ZnO/SPCE under different additions of RhB from 50 μM to 500 μM to 0.05 M PBS (pH 7) at 0.1 V/s and (**B**) Corresponding linear plot. (**C**) Voltammetric curves of Ag-3/ZnO/SPCE under different scan rates from 0.02–0.2 V/s and (**D**) corresponding linear plot.

The impact of concentration in the electrochemical detection of RhB using Ag-3/ZnO/SPCE was analyzed based on the CV technique by consecutively adding different amounts of RhB (50–500 μM) to 0.05 M PBS (pH 7) at 0.1 V/s. The resultant CV curves are presented in Figure 6A. Analyzing these curves, it was clear that the peak current increased linearly with the RhB concentration. It was further noted that this increase in the oxidation peak current was almost at the same rate for the different concentrations. The corresponding linear plot is given in Figure 6B; the resultant regression equation is *I*_pa_ = 0.28x + 45.65 (R^2^ = 0.9991). From this steady increase in the oxidation peak current with the RhB concentration, we confirmed that Ag-3/ZnO/SPCE was excellent in the electrochemical detection of RhB.

The scan rate impact in RhB determination was evaluated as part of this study. In this experiment, the CV technique was utilized to record the current responses of Ag-3/ZnO/SPCE in 0.05 M PBS (pH 7), comprising 100 μM RhB under different scan rates (0.02–0.2 V/s). The resultant voltammetric responses are given in Figure 6C. A balanced linear rise in the peak current with an increase in the scan rate can be clearly noticed in this figure. The regression equation calculated from the corresponding linear plot in Figure 6D is *I*_pa_ = 0.376*x* + 36.91 (μA, mV s^−1^, R^2^ = 0.9986). To determine the process involved in the electrochemical reaction of RhB at Ag-3/ZnO/SPCE, log (I_pa_) against log (scan rate) was further evaluated; it affirmed that the electrochemical process is adsorption controlled [30].

#### 3.2.3. Electrochemical Determination of RhB Based on DPV

**Figure 7:**
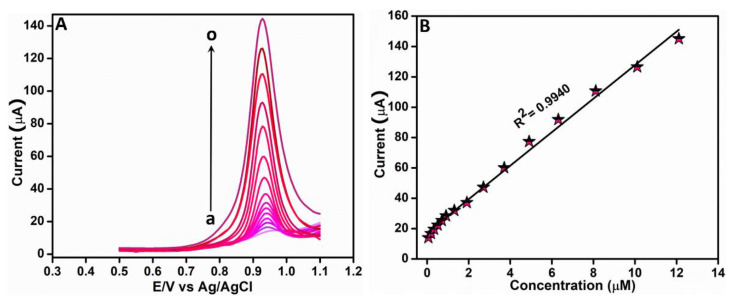
(**A**) DPV curves of Ag-3/ZnO/SPCE under different RhB concentrations in 0.05 M PBS (pH 7). (**B**) Linear plot of peak currents vs. RhB concentrations.

In this work, the DPV technique was utilized for the sensitive determination of RhB. The differential pulse voltammograms of Ag-3/ZnO/SPCE under various RhB concentrations in 0.05 M PBS (pH 7) were recorded; the resultant curves are shown in Figure 7A. These results showed a steady increase in the oxidation current with the RhB concentration; the peak potential remained stable even as the RhB concentration increased. The regression equation determined from the corresponding linear plot in Figure 7B is I (µA) = 11.04x + 17.304 (R^2^ = 0.9940). A very high sensitivity of 151.44 µAµM^−1^cm^−2^ and low limit of detection (LOD) of 0.8 nM towards RhB detection in the linear range 0.06–12.11 µM were exhibited by the proposed sensor. The LOD was calculated from the linear plot using the equation given below.

LOD = 3 S/b(2)

where S is the standard deviation and b is the slope attained from the linear calibration plot shown in Figure 7B. From this DPV analysis, which resulted in exceptional sensitivity, ultra-low LOD, and broad linear range, we affirmed that Ag-3/ZnO/SPCE is excellent in RhB detection. Table 1 shows the comparison of the results attained from this work with already reported works on the electrochemical detection of RhB.

#### 3.2.4. Selectivity, Stability, Reproducibility, and Repeatability Analysis

**Figure 8:**
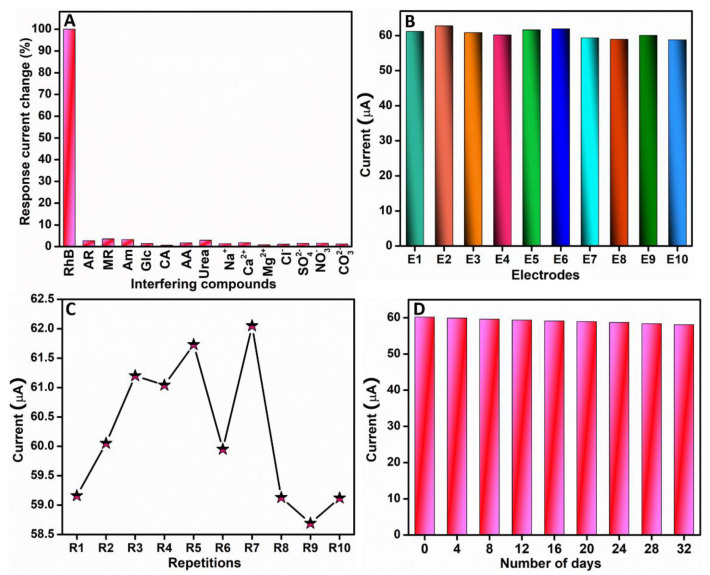
Analysis of (**A**) selectivity, (**B**) reproducibility, (**C**) repeatability, and (**D**) stability of proposed RhB sensor.

Interference is a major issue that hinders the reliability of an electrochemical sensor. Interference issues arise during sensing when concomitant species exist; a reliable electrochemical sensor must possess appreciable anti-interference. Hence, the selectivity property of the RhB sensor was analyzed using the DPV method. In this experiment, the peak current of RhB in the existence of a 10-fold excess concentration of a common interfering species including Allura Red (AR), methyl red (MR), amaranth (Am), glucose (Glc), citric acid (CA), ascorbic acid (AA), urea, 200-fold sodium (Na^+^), calcium (Ca^2+^), magnesium (Mg^2+^), chloride (Cl^−^), sulfate (${\mathrm{SO}}_{4}^{2-}$), carbonate (${\mathrm{CO}}_{3}^{2-}$), and nitrate (${\mathrm{NO}}_{3}^{-}$) was analyzed. The resultant variation in the response current from this anti-interference analysis conducted in 0.05 M PBS (pH 7), as in Figure 8A, showed that a just negligible variation occurred in the RhB peak current response in the existence of the aforementioned species. The maximum error in the response current of RhB observed was only 3.5%, which is inconsequential. Therefore, we confirmed that the proposed RhB sensor exhibited an outstanding anti-interference property.

The reproducibility of the proposed RhB sensor was evaluated by fabricating 10 identical Ag-3/ZnO/SPCEs and recording their respective differential pulse voltammetric responses in the presence of RhB in 0.05 M PBS (pH 7). The results are shown in Figure 8B. It was evident that only minor variations existed among the peak currents of all these fabricated electrodes. Further, we evaluated the relative standard deviation (RSD) of the peak currents we obtained for all the 10 modified electrodes and found it to be 2.19%, which is very low. Hence, from the aforementioned observations, it was evident that the proposed RhB sensor evinced an outstanding reproducibility. The experiment was conducted to evaluate the repeatability of the RhB sensor. As part of this experiment, differential pulse voltammetric responses of the proposed RhB sensor in 0.05 M PBS (pH 7) were taken 10 times, and the peak currents were noted. Figure 8C depicts the resultant peak current values of the proposed sensor towards RhB detection, and the RSD was a mere 2.01%. From the aforementioned inferences, we confirmed that the RhB sensor exhibited a good repeatability property. Further, the experiment was conducted for a period of 32 days to analyze the stability of the developed RhB sensor. The variation in the differential pulse voltammetric responses of the proposed sensor 0.05 M PBS (pH 7) was stringently observed during this period; the results are shown in Figure 8D. Only a negligible peak current response variation of 3.51% was observed at the end of 32 consecutive days when compared with the first day. This negligible variation in the peak current response indicated a good stability of the developed RhB sensor.

#### 3.2.5. Real Sample Analysis

The practical feasibility of the RhB sensor using Ag-3/ZnO/SPCE was evaluated in this work by conducting a real sample analysis based on the DPV technique. Red chili sauce, tomato juice, and paprika, obtained from a local supermarket, were selected as the real samples in this experiment; the presence of RhB was examined using a standard addition method. Table 2 shows the results we obtained from this experiment in the determination of RhB from the aforementioned real samples. The recovery values and RSD of the three measurements of peak currents from all the resultant voltammetric responses were calculated. It was observed that the RhB sensor exhibited excellent recovery values for all the real samples, between 96.66–103.83%, and a maximum RSD of just 2.96%. Therefore, by conducting this investigation, an excellent practical feasibility of the RhB sensor was confirmed.

### 3.3. Photocatalytic Degradation of RhB

**Figure 9:**
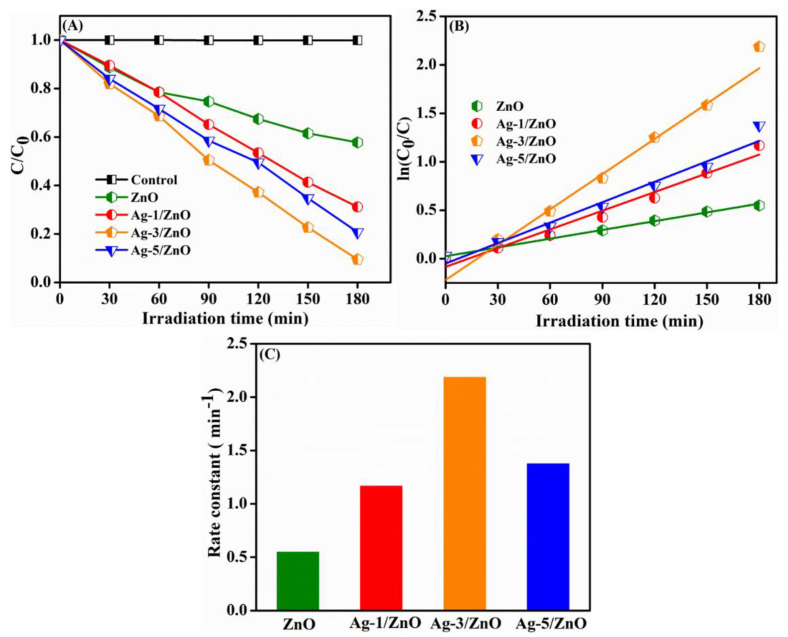
Photodegradation of RhB (**A**), corresponding pseudo-first-order kinetic plots (**B**), and rate constant plot (**C**) of ZnO and Ag/ZnO.

To examine the photocatalytic activity of Ag/ZnO, RhB was used as the model pollutant in the existence of visible light irradiation. The photocatalytic activity and kinetics of pure ZnO, Ag-1/ZnO, Ag-3/ZnO, and Ag-5/ZnO for the degradation of RhB under visible light are illustrated in Figure 9. A control experiment (without catalyst) was also conducted and is shown in the same figure for comparison purposes. We measured the absorption of RhB at 554 nm with different intervals of time to investigate the degradation process. Figure 9A represents the RhB degradation rate in the existence of various catalysts. In the control experiment, *C*/*C*_0_ versus a time curve indicated only 0.05% RhB was degraded after 180 min of visible light irradiation, owing to the high stability of RhB in the presence of light. The 3D, hierarchical, nanosheet-based, ZnO flower-like nanostructure exhibited 42.2% of RhB degradation. When we compared the degradation of RhB using different wt% of Ag on ZnO, Ag-3/ZnO obtained 90.5% of degradation while the degradation percentages of Ag-1/ZnO and Ag-5/ZnO were 69.1% and 75.01%, respectively. 

The experimental results suggest that the decoration of Ag NPs on the ZnO surface enhanced the visible light photocatalytic degradation of RhB as a result of the excellent capability of separating photogenerated charge carriers, large specific surface area, and better light absorption [38]. The photocatalytic degradation of RhB followed a pseudo-first-order reaction; the equation is given as $ln(C_{0}/C)=kt$. Figure 9B represents the fitted kinetic curve, *ln*(*C*_0_/*C*) versus time of the hierarchical ZnO and Ag-decorated ZnO. Usually, the k value can be evaluated as the photocatalytic activity; it is obtained from the slope of the kinetic curve. The degradation rate constant of the ZnO and Ag-modified ZnO are illustrated in Figure 9C. It is noticeable that all Ag-modified nanocomposites obtained higher rate constant values than the pure ZnO. The Ag-3/ZnO achieved a higher k value, 2.18 min^−1^, than the other nanocomposites, which was, indeed, four times higher than ZnO (0.548 min^−1^). The rate constant values of the Ag-1/ZnO and Ag-5/ZnO nanocomposite were 1.166 and 1.375 min^−1^, respectively. The higher photocatalytic effect of Ag-3/ZnO was described by the presence of the surface plasmon resonance effect (SPR) of the Ag NPs that facilitated the e–h pair separation by the inhibition of the charge recombination. A lower amount of Ag reduced the scattering of light, which made it a superior photocatalyst. A higher amount of Ag NPs on ZnO led to the agglomeration of particles, resulting in the reduction in the surface area for effective light absorption. In addition, the higher amount of Ag on ZnO covered the surface from light absorption and acted as the recombination center by the electrostatic attraction between Ag+ and holes, leading to the unavailability of an appropriate place for the additional formation of the e–h pair. Therefore, the higher amount of Ag on ZnO showed an inhibited photocatalytic activity.

## 4. Conclusions

In summary, we successfully developed an ultrasensitive electrochemical sensor for the nanomolar-level detection of the organic dye RhB based on a dual-functional, Ag-3/ZnO-modified SPCE. Electrochemical investigations carried out using the CV and DPV techniques revealed the excellent electrocatalytic activity of the proposed Ag-3/ZnO/SPCE towards RhB detection. A very high sensitivity and low detection limit were observed for the RhB sensor; its reliability was verified by conducting different electrochemical experiments. Moreover, the prepared nanomaterial is a promising photocatalyst for the effective degradation of RhB. It is noticeable that the presence of Ag NPs enhanced the absorption from the visible region and suppressed the electron-hole pair recombination through the SPR effect of Ag NPs. Further, 3 wt% of the Ag-modified ZnO nanocomposite exhibited the highest photodegradation efficacy (90.5%) in the presence of visible light. This excellent photocatalytic activity of Ag/ZnO is associated with the synergetic effect of Ag NPs and hierarchical ZnO nanomaterials.

## Figures and Tables

**Table 1:** Comparison of performance of proposed modified electrode in RhB detection.

Modified Electrode	Detection Technique	LOD (µM)	Linear Range (µM)	pH	References
MnO2NRs-ERGO/GCE	SDLSV	2.87	9.58–479; 479–9580	6	[31]
Cu@CS/GCE	DPV	0.1	0.3–30	6.5	[32]
MWCNT/CPE	DPV	0.02	0.1–15	3	[33]
MWCNT-PEI/GCE	SDLSV	0.006	0.01–10	6.3	[34]
Nd-MOF/GCE	DPV	0.0036	0.08–2.0; 2.0–4.0	6	[35]
GS/GCE	DPV	0.0015	0.005–0.12	6.5	[36]
MWCNT-COOH/IL/PGE	DPV	0.001	0.005–2.0; 2.0–60.0	5	[37]
Ag-3/ZnO/SPCE	DPV	0.0008	0.06–12.11	7	This work

Abbreviations: MWCNT, multi-walled carbon nanotubes; IL, ionic liquid; PGE, pencil graphite electrode; Nd, neodymium; MOF, metal-organic framework; GCE_,_ glassy carbon electrode; ERGO, electro-reduced graphene oxide; SDLSV, second-order derivative linear scan voltammetry; PEI, polyethylenimine; GS, graphene nanosheets; CPE, carbon paste electrode; CS, carbon sphere.

**Table 2:** Determination of RhB in real samples using proposed sensor.

Sample	Added (µM)	Found (µM)	Recovery (%)	RSD ^k^
Red chili sauce	2	2.01	100.83	2.73
	4	4.09	102.25	2.96
	8	7.87	98.37	1.87
Tomato juice	2	1.97	98.5	2.21
	4	4.11	102.83	2.02
	8	7.99	99.87	2.08
Paprika	2	1.93	96.66	2.94
	4	4.15	103.83	1.96
	8	7.86	98.25	1.59

^k^: RSD of three measurements.

## Data Availability

Not applicable.
